# Isolation of *Clostridium botulinum* type C from a wound in a pig

**DOI:** 10.1177/10406387251330885

**Published:** 2025-04-10

**Authors:** Benedetta Cordioli, Manuel Garbuio, Eliana Schiavon, Alessia Rizzardi, Angela Guolo, Serena Genovese, Ilenia Drigo, Luca Bano

**Affiliations:** Diagnostic and Microbiology Laboratory, Istituto Zooprofilattico Sperimentale delle Venezie, Villorba, Veneto, Italy; Diagnostic and Microbiology Laboratory, Istituto Zooprofilattico Sperimentale delle Venezie, Villorba, Veneto, Italy; Legnaro, Veneto, Italy; Diagnostic and Microbiology Laboratory, Istituto Zooprofilattico Sperimentale delle Venezie, Villorba, Veneto, Italy; Diagnostic and Microbiology Laboratory, Istituto Zooprofilattico Sperimentale delle Venezie, Villorba, Veneto, Italy; Diagnostic and Microbiology Laboratory, Istituto Zooprofilattico Sperimentale delle Venezie, Villorba, Veneto, Italy; Diagnostic and Microbiology Laboratory, Istituto Zooprofilattico Sperimentale delle Venezie, Villorba, Veneto, Italy; Diagnostic and Microbiology Laboratory, Istituto Zooprofilattico Sperimentale delle Venezie, Villorba, Veneto, Italy

**Keywords:** *Clostridium botulinum* type C, pigs, wound

## Abstract

We report here the isolation of *Clostridium botulinum* type C from the medial muscles of the thigh of a gestating gilt with a claw wound and without evidence of septicemia. The pig died with paralytic signs, consistent with wound botulism, similar to episodes in humans.

Botulism is a neuroparalytic disease caused by botulinum neurotoxins (BoNTs), which are produced by several distinct clostridial species and are classified into 7 types (A–G); C and D and the mosaic variants C/D and D/C are the most frequent types involved in animal botulism.^8^ Outbreaks occur frequently in cattle and poultry, whereas pigs are considered resistant.^14^ Indeed, the few reports that describe the disease in pigs all involved or suspected the ingestion of pre-formed toxin via feed or carcasses.^4,7,9,14^

Botulism can also occur in toxicoinfectious forms, in which bacteria replicate in the gut (intestinal or infant botulism) or damaged tissues (wound botulism) and produce BoNTs.^11^ This disease mechanism has been described in humans but not in pigs, to our knowledge. We retrieved no cases of isolation of *Clostridium* (*C.*) *botulinum* from pig wounds in a search of Google, PubMed, CAB Direct, Web of Science, and Scopus, using the search terms “isolation”, “botulinum”, “pig”, and “wound”, suggesting that this condition has not been reported previously in swine. For this reason, we report here the isolation of *C. botulinum* type C from the thigh muscles of a gestating gilt with a claw wound.

In a sow farm of ~375 sows located in Padua, Italy, a gestating gilt in the second third of pregnancy was weak and became laterally recumbent and paralyzed, as reported by the practitioner. The gilt was the only animal affected in the barn, making it difficult to add further clinical information on the paralytic features. The animal did not receive antibiotics and died 3 d after the appearance of clinical signs.

We performed an autopsy on-site within 6 h of the death of the animal, and noted a mild medial claw wound on the right hind foot. After skinning, the claw lesion was observed to extend to the subcutaneous tissues and muscles, reaching the medial muscle plane of the thigh, with irregular edges, brown-red exudate, and fetid odor. Thoracic and the abdominal organs were mildly autolytic but were without gross lesions. The presumptive diagnosis was severe subacute necrotic bacterial myositis.

Samples of thigh muscle and femoral bone marrow were collected aseptically, plated on blood agar (BA), eosin–methylene blue (EMB), and perfringens agar base (PAB), and incubated aerobically (BA, EMB) and anaerobically (BA, PAB) at 37°C for 24–48 h. In addition, we inoculated samples into fortified cooked meat medium (FCMM); samples were heat-shocked and then incubated at 37°C in anaerobic conditions to select spore-forming bacteria. After 48 h, we plated FCMM on differential agar media incubated aerobically and anaerobically for 48 h. Aerobic plates (BA, EMB) served as heat-shock control. BA base modified as described elsewhere^2^ (BAB2), phenylethyl alcohol agar (PEAA), PAB, egg yolk agar (EYA), and BA were plated to detect anaerobic bacterial strains. Bacterial cultures were identified by matrix-assisted laser desorption/ionization time-of-flight mass spectrometry (MALDI TOF MS; Bruker), and the database was implemented with *C. botulinum* group III profiles.^3^

The bacteriologic examination of bone marrow was negative; muscle cultures were positive for *Pasteurella multocida*, *Streptococcus dysgalactiae*, *Escherichia fergusonii*, *C. perfringens*, *C. tertium*, and *C. botulinum* group III (Table 1). We tested the actual BoNT production of the *C. botulinum* isolate using a mouse bioassay conducted on the filtered supernatant of FCMM broth previously inoculated with the pure isolate^5^; this procedure was approved by the Ethics Committee of the Istituto Zooprofilattico Sperimentale delle Venezie (opinion 24/2014) and authorized by the Italian Ministry of Health (authorization 243/2020-PR). In addition, we performed a quantitative real-time PCR (qPCR) targeted on the BoNTs encoding genes on FCMM,^1^ which revealed the presence of *C. botulinum*–producing non-mosaic BoNT type C.

**Table 1.** Results of classical bacteriology and FCMM examinations on femoral bone marrow and muscle.

Sample	Classical bacteriology	FCMM
Femoral bone marrow	Negative	Negative
Muscle	*Pasteurella multocida*	*Clostridium perfringens*
	*Escherichia fergusonii*	*Clostridium tertium*
	*Streptococcus dysgalactiae*	*Clostridium botulinum* type C
	*Clostridium perfringens*	
	*Clostridium tertium*	

FCMM = fortified cooked meat medium.

Because bacterial examination from the bone marrow was negative, septicemia caused by multiple bacteria species was considered unlikely. The concurrent isolation of *C. perfringens* from the muscles may raise questions about a possible case of gas gangrene or clostridial myonecrosis, as described in humans. Histopathology was not performed, and this possibility cannot be fully excluded. However, even if the disease has been replicated in animal models, the role of *C. perfringens* type A in animal gas gangrene remains speculative.^13^ In addition, because we performed the autopsy within 6 h of death, it is unlikely that *C. botulinum* grew postmortem. Therefore, *C. botulinum* may have reached the muscle from the wound in the right hind foot and may have been producing BoNTs.

Few pig botulism cases have been reported, and type C has always been described in such cases, suggesting a higher prevalence of type C in swine; D/C and C/D are the serotypes more frequently reported in cattle and poultry, respectively.^4,7,8,9,14^

The diagnosis of botulism (even in humans) is based primarily on the detection of 1) BoNTs in sera, or 2) BoNT producing clostridia in biological samples (feces, wounds, intestinal content, liver, ruminal content, etc.) coupled with paralytic signs.^10^ Unfortunately, BoNTs were not directly investigated in the serum in our case, as we could not perform serum sampling before the gilt died. However, the isolation of the toxigenic strain combined with the clinical presentation led to the hypothesis of wound botulism.

This toxicoinfectious form of botulism has not been reported in swine, to our knowledge. Its occurrence likely depends on the presence of *C. botulinum* type C in the digestive tract of pigs, resulting in environmental contamination.^9^ Additionally, because *C. botulinum* spores have been detected in the livers of healthy pigs, the possibility of hematogenous dissemination through the gastrointestinal tract, described for other clostridia (e.g., *C. chauvoei*), cannot be excluded.^15^

Only one study conducted in Japan specifically assessed the prevalence of *C. botulinum* type C in pig farms, reporting positive results in livers (8–100%) and cecal contents (80%) of healthy animals.^15^ Research has focused on the genes encoding for *C. botulinum* toxins type A, B, and F (involved in human botulism) in pig feces and intestinal samples collected at slaughter, with prevalence reported as 4–62%.^6,12^

Botulism could be underestimated in swine pathology, and further studies on its prevalence may be useful in evaluating whether it should be included in the differential diagnosis of paralytic syndromes observed in pigs with wounds.
